# Swine Influenza Virus Antibodies in Humans, Western Europe, 2009

**DOI:** 10.3201/eid1703100581

**Published:** 2011-03

**Authors:** Nancy A. Gerloff, Jacques R. Kremer, Emilie Charpentier, Aurélie Sausy, Christophe M. Olinger, Pierre Weicherding, John Schuh, Kristien Van Reeth, Claude P. Muller

**Affiliations:** Author affiliations: Centre de Recherche Public de la Santé/Laboratoire National de Santé, Luxembourg, Grand-Duchy of Luxembourg (N.A. Gerloff, J.R. Kremer, E. Charpentier, A. Sausy, C.P. Muller);; Laboratoires Réunis, Junglinster, Luxembourg (C.M. Olinger, J. Schuh);; Health Directorate, Luxembourg (P. Weicherding);; Ghent University, Ghent, Belgium (K. Van Reeth)

**Keywords:** Swine, neutralizing antibodies, pandemic (H1N1) 2009, avian-like H1N1 swine influenza virus, Europe, influenza, viruses, research

## Abstract

Serologic studies for swine influenza viruses (SIVs) in humans with occupational exposure to swine have been reported from the Americas but not from Europe. We compared levels of neutralizing antibodies against 3 influenza viruses—pandemic (H1N1) 2009, an avian-like enzootic subtype H1N1 SIV, and a 2007–08 seasonal subtype H1N1—in 211 persons with swine contact and 224 matched controls in Luxembourg. Persons whose profession involved contact with swine had more neutralizing antibodies against SIV and pandemic (H1N1) 2009 virus than did the controls. Controls also had antibodies against these viruses although exposure to them was unlikely. Antibodies against SIV and pandemic (H1N1) 2009 virus correlated with each other but not with seasonal subtype H1N1 virus. Sequential exposure to variants of seasonal influenza (H1N1) viruses may have increased chances for serologic cross-reactivity with antigenically distinct viruses. Further studies are needed to determine the extent to which serologic responses correlate with infection.

Pandemic (H1N1) 2009 influenza virus resulted from genetic reassortment between at least 2 swine influenza viruses (SIVs) (*1*). Hemagglutinin (HA) of this novel subtype H1N1 virus is similar to that of classical swine influenza virus and the triple reassortant subtype H1N1 viruses that are endemic in swine populations in North America. At the time of its detection in humans, pandemic (H1N1) 2009 virus had never been detected in swine populations anywhere, but it is believed to have circulated undetected in regions with little or no surveillance for influenza viruses in swine. Because this virus has not been reported by the European Surveillance Network for Influenza in Pigs (www.esnip.ugent.be) since the network’s inception in 2001, it was most likely absent in swine in western Europe. By the end of 2009, pandemic (H1N1) 2009 virus infection of swine had been reported in Norway (*2*); sporadic cases have been reported in a few other European countries (e.g., Germany, Italy, Denmark) (*3*). The swine were probably infected by contact with infected humans, whereas transmission from swine to humans has not yet been documented. Pandemic (H1N1) 2009 virus is the first swine-origin virus that is readily transmitted between humans (*4*).

Human infections with SIVs are rare. During 1958–2005, only 50 cases of zoonotic infections were reported; most were in persons who had contact with swine (*5*). Limited secondary transmission to close contacts has been reported but appears to be rare, and to our knowledge, sustained human-to-human transmission of enzootic SIVs has never been noted (*6*). Some serologic studies suggest that persons who work with swine are at increased risk for zoonotic infection with SIVs (*7**–**12*).

The predominant subtype H1N1 SIVs in Europe were introduced from wild ducks to swine in 1979 and have an entirely avian-derived genome (*13**–**15*). These viruses are designated as avian-like viruses and are antigenically distinct from subtype H1N1 SIVs in North America and from pandemic (H1N1) 2009 virus. Few cases of human infection with these avian-like swine subtype H1N1 viruses have been reported; chains of transmission have not been found (*5**,**9**,**15*), and no serologic studies have provided indirect evidence of transmission of SIVs to humans in Europe (*15*).

Studies in the United States, United Kingdom, and Finland found antibodies against pandemic (H1N1) 2009 virus in elderly persons (*16**–**18*). These antibodies can be explained by antigenic evolution of seasonal human influenza (H1N1) viruses that are derived from the 1918 pandemic virus (such as the classical swine influenza [H1N1] virus) but have undergone greater antigenic drift than the swine virus (*19*). Antigenically, the influenza (H1N1) viruses that circulated among humans before the 1950s are probably more closely related to the classical swine virus and thus to the pandemic (H1N1) 2009 virus than to contemporary human subtype H1N1 viruses. We investigated whether persons whose professions involve contact with swine (swine workers [SWs]) have neutralizing antibodies against 3 influenza viruses: pandemic (H1N1) 2009 virus, a European avian-like subtype H1N1 SIV, and a 2007–08 seasonal influenza subtype H1N1 (seasonal influenza) virus.

## Methods

### Study Population

During July 20–28, 2009, blood was collected from 211 healthy persons with past or present professional contact with swine. All participants gave informed consent and completed a questionnaire about the nature of their swine contacts (occupation, duration, frequency), influenza vaccination, and influenza infection history. No participant reported having been infected with pandemic (H1N1) 2009 virus. A total of 224 control serum samples were obtained from the serum bank of the Laboratoires Reunis, Junglinster, Luxembourg. The samples, from the general population of Luxembourg, had been submitted in December 2008 for routine serologic testing. Because of ethical constraints, no further information was gathered from controls. The study was approved by the National Ethical Committee for Research in Humans.

### Virus Neutralization Assay

According to recommended World Health Organization protocols (*20*), serum samples were tested by virus neutralization assay against an influenza A (H1N1) virus strain isolated from a patient in Luxembourg in July 2009 (A/Luxembourg/43/2009). Complete genome analyses revealed that the sequence was almost identical to the prototype vaccine virus (A/California/7/2009) and represented a typical North American/European pandemic (H1N1) 2009 virus (*4*). Nucleotide sequences are available from GenBank (accession nos. FN423708–15). A/swine/Belgium/1/98 is representative of the avian-like subtype H1N1 SIVs that are enzootic in swine populations of western Europe (*21*). Both viruses have an antigenically distinct H1 and ≈72% aa identity in the HA1 region (93% and 98% aa identity in neuraminidase [NA] and matrix [M] proteins) (*22*). A representative of the 2007–08 seasonal influenza virus was included in the assay and had 73% and 74% identity in HA1 proteins compared with pandemic (H1N1) 2009 virus and SIV (A/Luxembourg/572/2008 HA gene, accession no. FR716024).

Positive control serum was collected from 5 patients >5 weeks after recovery from a laboratory-confirmed infection with pandemic (H1N1) 2009 virus and from a previously unexposed pig 4 weeks after it had been experimentally infected with A/swine/Belgium/1/98 (H1N1) (*21*). Before the assay was conducted, all samples were heated to 56°C for 30 min to inactivate complement and unspecific inhibitors. Titers were reported as the reciprocal of the highest dilution of serum that completely neutralized virus growth. Samples were first screened in duplicate with a 1:10 dilution. All samples that showed virus neutralization in ≥1 well were further titrated in quadruplicate up to a dilution of at least 1:320. Control samples positive for both viruses were included in all assays.

### Statistical Analyses

Geometric mean titers (GMTs) were calculated for each person from quadruplicate serum samples. All negative samples were given an arbitrary GMT of 5. GMTs were compared by using the nonparametric Wilcoxon rank-sum test. To examine bivariate risk factors associated with antibody prevalence, we dichotomized GMTs of all positive samples for different cutoff points (>10 to >80) and analyzed them by χ^2^ test and, for low proportions, by *z*-test. The distribution of antibody levels was checked for associations with multiple risk factors by using proportional odds modeling (*23**,**24*). Statistical analyses were performed by using SigmaStat version 3.1 (San Jose, CA, USA) and SPSS version 18 (Chicago, IL, USA).

## Results

### Study Population

Mean age of the 211 SWs was 48.2 years (range 18–94 years); 67.8% were male (Table 1). Most (84.8%) SWs reported having worked daily in close contact with swine (distance <1 m, 83%) for >10 years (73.5%). Among the SWs, 133 were involved in pig breeding, fattening, or general pig farming; 51 were slaughterhouse workers; 12 were veterinarians; 13 were butchers; and 2 were hunters. The 224 controls were matched with SWs by age and sex (Table 1).

**Table 1:** Characteristics of persons tested for 3 influenza viruses, Luxembourg, 2008–2009*

Characteristic	Swine workers, no. (%),† n = 211	Controls, no. (%),‡ n = 224
Sex		
M	143 (67.8)	151 (67.4)
F	68 (32.2)	73 (32.6)
Age group, y§		
18–40	69 (32.7)	80 (35.7)
41–50	59 (28)	58 (25.9)
51–60	39 (18.5)	41 (18.3)
61–94	44 (20.9)	45 (20.1)
Profession		
Farmer	133	NA
Slaughterhouse worker	51	NA
Other¶	27	NA
Years worked with swine		
<1	4 (1.9)	NA
1–4	26 (12.3)	NA
5–10	26 (12.3)	NA
>10	155 (73.5)	NA
Unknown	0	224
Frequency of swine contact		
Rarely	3 (1.4)	NA
Monthly	2 (0.9)	NA
Weekly	25 (11.8)	NA
Daily	179 (84.8)	NA
Unknown	2 (0.9)	NA
Frequency of close contact (<1 m) with swine		
Never	1 (0.5)	NA
Rarely	3 (1.4)	NA
Occasionally	10 (4.7)	NA
Often	22 (10.4)	NA
Always	175 (82.9)	NA
Self-reported influenza vaccine in past 5 y		
No/unsure	155 (73.5)	NA
Yes	56 (26.5)	NA
Self-reported infection with seasonal influenza		
No	145 (68.7)	NA
Yes	57 (27.0)	NA
Exposure to swine		
Only until 1997	26	NA
Only until 2007	59	NA
Until time of collection	152	NA

*NA, not available. †Sampled in July 2009. ‡Sampled in December 2008. §Mean (median) age 48.2 (48) years for swine workers, 47.6 (47.2) years for controls. ¶Veterinarian, butcher, hunter.

### Antibodies against Pandemic (H1N1) 2009 Virus

GMTs of antibodies against pandemic (H1N1) 2009 virus (Table 2) were significantly higher for SWs than for controls (p = 0.004). Table 3 shows that 2× more SWs than controls had neutralizing antibodies against pandemic (H1N1) 2009 virus for the lowest cutoff value (p = 0.001). This ratio slightly increased with rising cutoff values and remained significant to a cutoff >160 (Table 3). In all age groups, ≈2× more SWs than controls had antibodies against pandemic (H1N1) 2009 virus (cutoff >10), except for persons >60 years of age (Table 4). For SWs and controls >60 years of age, GMTs for pandemic (H1N1) 2009 virus were similar (p = 0.897; Table 2). GMTs were significantly higher for younger than for older (>60 years) SWs (but not controls) (Table 2). Among SWs, antibodies against pandemic (H1N1) 2009 virus tended to decrease with age for all cutoff values; among controls, the same was observed for cutoffs >10 to >40. Thus, younger SWs more often had higher levels of antibodies against pandemic (H1N1) 2009 virus than did controls and older SWs. The difference between SWs and controls disappeared in older age groups and was weaker when older and younger controls were compared.

**Table 2:** Geometric mean titers for 3 influenza viruses in swine workers and controls, Luxembourg, 2008–2009*

Virus (strain) and participant age, y	Study sample, % (95% CI)		p value†
	Swine workers, n = 211	Controls, n = 224	
Pandemic (H1N1) 2009 (A/Luxembourg/43/2009)			
All	8.7 (7.5–10)	6.1 (5.6–6.6)	**0.004**
<60	9.2 (7.6–11.1)‡	6 (5.5–6.7)	**<0.05**
>60	5.6 (4.5–6.9)	5.4 (4.6–6.4)	0.897
Avian-like SIV (H1N1) (A/swine/Belgium/1/98)			
All	10.3 (8.8–12)	7.7 (6.9–8.5)	0.168
<60	9.8 (8.1–11.8)	6.4 (5.8–7)§	**<0.05**
>60	11.2 (8–15.5)	13.6 (9.9–18.5)	0.170
Seasonal influenza (H1N1) (A/Luxembourg/572/2008)¶			
All	23.2 (20.3–26.4)	13.9 (12.1–15.9)	**<0.001**
<60	21.3 (18.3–24.7)	12.4 (10.7–14.4)#	**<0.001**
>60	30.6 (22.7–41.1)	20.1 (15.3–28.7)	0.083

*%, no. persons/total no. persons in age groups with geometric mean titer cutoff >10. SIV, swine influenza virus. †p value <0.05 for significance were calculated by using the Wilcoxon rank-sum test. **Boldface** indicates significance (p<0.05). ‡p<0.05, compared with swine contacts of the age group >60 y against pandemic (H1N1) 2009 virus. §p<0.001, compared with controls of the age group >60 y against avian-like SIV. ¶Data for 210 swine workers, 221 controls. #p = 0.001, compared with controls of the age group >60 y against seasonal influenza (H1N1).

**Table 3:** Neutralizing antibody reactivity against 3 influenza viruses in swine workers and controls, Luxembourg, 2008–2009*

Virus (strain) and cutoff value	Swine workers, no. (%; 95% CI), n = 211	Controls, no. (%; 95% CI), n = 224	p value
Pandemic (H1N1) 2009 (A/Luxembourg/43/2009)			
>10	46 (21.8; 16.8–27.9)†	23 (10.3); 6.9–14.9)‡	**0.001§**
>20	37 (17.5; 13–23.2)†	16 (7.1; 4.4–11.3)‡	**0.001§**
>40	31 (14.7; 10.6–20.1)	12 (5.4; 3.1–9.1)	**0.002§**
>80	14 (6.6; 4–10.8)	4 (1.8; 0.7–4.5)	**0.02§**
>160	6 (2.8; 1.3–6.06)	0 (0; 0–1.2)	**0.033¶**
>320	5 (2.4; 1–5.4)	0 (0; 0–1.2)	0.061¶
Avian-like SIV (H1N1) (A/swine/Belgium/1/98)			
>10	66 (31.3) 25.4–37.8)	59 (26.3; 21–32.5)	0.289§
>20	57 (27; 21.5–33.4)	38 (17; 12.6–22.4)	**0.015§**
>40	39 (18.5; 13.8–24.3)	12 (5.4; 3.1–9.1)	**<0.001§**
>80	21 (10; 6.6–14.7)	4 (1.8; 0.7–4.5)	**<0.001§**
>160	9 (4.3; 2.3–7.9)	1 (0.4; 0.1–2.5)	**0.019¶**
>320	4 (1.9; 0.7–4.8)	1 (0.4; 0.1–2.5)	0.331¶
Seasonal influenza (H1N1) (A/Luxembourg/572/2008)#			
>10	183 (87.1; 83–91.9)	132 (59.7; 53.2–66)	**<0.001§**
>20	125 (59.5; 52.8–65.9)	76 (34.4; 28.4–40.9)	**<0.001§**
>40	61 (29; 23.3–35.5)	39 (17.6; 13.2–23.2)	**0.007§**
>80	21 (10; 6.6–14.9)	17 (7.7; 4.8–12)	0.500§
>160	14 (6.7; 3.9–11)	7 (3.2; 1.4–6.5)	0.144§
>320	11 (5.2; 2.9–9.2)	4 (1.8; 0.5–4.7)	0.093§

*Values are no. persons with antibodies. CI, confidence interval; SIV, swine influenza virus. **Boldface** indicates significance (p<0.05). †p<0.05 when compared with swine workers against avian-like SIV (H1N1) of the same titer cutoff. ‡p<0.003 when compared with swine workers against avian-like SIV (H1N1) of the same titer cutoff. §χ^2^ test on 2-way table. ¶*z*-test. #Data for 210 swine workers, 221 controls.

**Table 4:** Neutralizing antibody reactivity >10 for 3 influenza viruses in swine workers and controls, Luxembourg, 2008–2009*

Participant age in 2009, y	Pandemic (H1N1) 2009 (A/Luxembourg/43/2009)			Avian-like SIV (H1N1) (A/swine/Belgium/1/98)			Seasonal influenza (H1N1) (A/Luxembourg/572/2008)	
	Swine workers	Controls		Swine workers	Controls		Swine workers	Controls
<40	22/69 (31.9; 22.1–43.6)†	12/80 (15; 8.8–24.4)		19/69 (27.5; 18.4–39.0)	15/80 (18.8; 11.7–28.7)		58/68 (85.3; 6.9–93.7)	50/78 (64.1; 53.5–74.8)
41–50	10/59 (16.9; 9.5–28.5)	5/58 (8.6; 3.7–18.6)		11/59 (18.6; 10.7–30.4)	8/58 (13.8; 7.2–24.9)		46/59 (78; 67.4–88.6)	29/57 (50.9; 37.9–63.9)
51–60	9/39 (15.3; 12.7–38.3)	3/41 (7.3; 2.5–19.4)		17/39 (43.6; 29.3–59.0)‡	8/41 (19.5; 10.2–34.0)		37/39 (94.9; 88.0–101.8)§	19/41 (46.3; 31.1–61.6)
>60	5/44 (8.6; 5.0–24.0)¶	3/45 (6.7; 2.3–17.9)#		19/44 (43.2; 29.7–57.8)	28/45 (62.2; 47.6–74.9)		42/44 (95.5; 89.3–101.6)**	34/45 (75.6; 63.0–88.1)
18–94 (total)	46/211 (21.8; 16.8–27.9)††	23/224 (10.3; 6.9–14.9)‡‡		66/211 (31.3; 25.4–37.8)	59/224 (26.3; 21.0–32.5)		183/210 (87.1; 83.0–91.9)	132/221 (59.7; 53.2–66.0)

*Values are no. persons/total no. persons in age groups with antibody reactivity >10 (%; 95% confidence interval). p values <0.05 cutoff for significance were calculated by using the χ^2^ test. SIV, swine influenza virus. †p = 0.012, compared with controls of the same age group against pandemic (H1N1) 2009. ‡p = 0.037, compared with controls of the same age group against avian-like SIV (H1N1). §p<0.001, compared with controls of the same age group against seasonal influenza (H1N1). ¶p = 0.002, compared with swine workers of the same age group against avian-like SIV (H1N1). #p<0.001, compared with controls of the same age group against avian-like SIV (H1N1). **p<0.05, compared with controls of the same age group against seasonal influenza (H1N1). ††p<0.05, compared with swine workers against avian-like SIV (H1N1). ‡‡p<0.001, compared with controls against avian-like SIV (H1N1).

### Antibodies against SIV

Similar to findings for pandemic (H1N1) 2009 virus, GMTs for SIV were higher among SWs than controls; however, the difference was not significant (Table 2; p = 0.168). More SWs than controls had positive SIV titers regardless of the cutoff (Table 3). These differences were significant for cutoffs >20 to >160 and increased with higher cutoffs (Table 3). Comparable to findings for pandemic (H1N1) 2009 virus, for age groups up to 60 years antibodies against SIV were found in 1.2–2× more SWs than controls (cutoff >10; Table 4); GMTs were significantly higher among SWs than controls in this age group (Table 2; p = 0.028). Seroprevalences and GMTs were similar for persons >60 years of age from each group (Tables 2, 4).

In contrast to findings for pandemic (H1N1) 2009 virus, the highest proportion of seropositive persons was found in older age groups, SWs >50 and controls >60 years (Table 4). GMTs were significantly higher among older (>60 years) than younger controls (p = <0.001) but differed little among SWs (Table 2; p = 0.293).

Thus, antibody titers for SIV were found more often and were higher among SWs than controls. In contrast to findings for pandemic (H1N1) 2009 virus, titers for SIV were found more often and were higher for older than younger controls; for SWs, titers were found more often among older persons but values were similar.

### Antibodies against Pandemic (H1N1) 2009 Virus and SIV

Among SWs, for all cutoff values seroprevalence was higher for SIV than for pandemic (H1N1) 2009 virus. The same was found for controls but only for lower titers (≥10 and ≥20; Table 3). The differences between antibody positivity for each of the 2 viruses increased with age among SWs and controls (Table 4). Comparing seroprevalences for pandemic (H1N1) 2009 virus to those for SIV, differences were significant only for SWs >60 years (p = 0.002). Also, significantly more controls of the same age group (>60 years) had antibodies against SIV (62.2%) than against pandemic (H1N1) 2009 virus (6.7%, p<0.001; Table 4). The proportion of older (>60 years) SIV-seropositive controls (62.2%) differed significantly from the proportion of younger (<60 years) SIV-seropositive controls (17.3%; p<0.001).

Thus, for both groups, more persons had antibodies against SIV than against pandemic (H1N1) 2009 virus, and differences in positivity decreased with increasing titers. Antibodies against SIV were more common among older persons, and antibodies against pandemic (H1N1) 2009 virus were more common among younger persons.

### Antibodies against SIV and Pandemic (H1N1) 2009 Virus

Antibody titers of convalescent-phase serum samples from patients with pandemic (H1N1) 2009 virus were 16× higher for pandemic (H1N1) 2009 virus than for SIV (GMTs 226.2 vs. 13.5, respectively), indicating low cross-reactivity between these viruses. Similarly, in a pig serum sample, GMT for SIV (>1,280) was 128× lower than that for pandemic (H1N1) influenza (*8*).

Among 66 SIV-positive serum samples from SWs, only 28 were also positive for pandemic (H1N1) 2009 virus (GMT cutoff >10). GMTs of at least single positive samples correlated significantly with each other (R^2^ = 0.5, correlation coefficient [CC] = 0.4, p<0.001; Figure, panel C); and GMTs for SIV were significantly higher than corresponding GMTs for pandemic (H1N1) 2009 virus (48, 95% CI 38.4–60.1, and 16.3, 95% CI 11.3–22.8, respectively; p<0.001). To the contrary, among SIV-positive controls GMTs for SIV did not correlate with GMTs for pandemic (H1N1) 2009 virus (R^2^<0.01, CC = 0.322; Figure, panel D).

**Figure:**
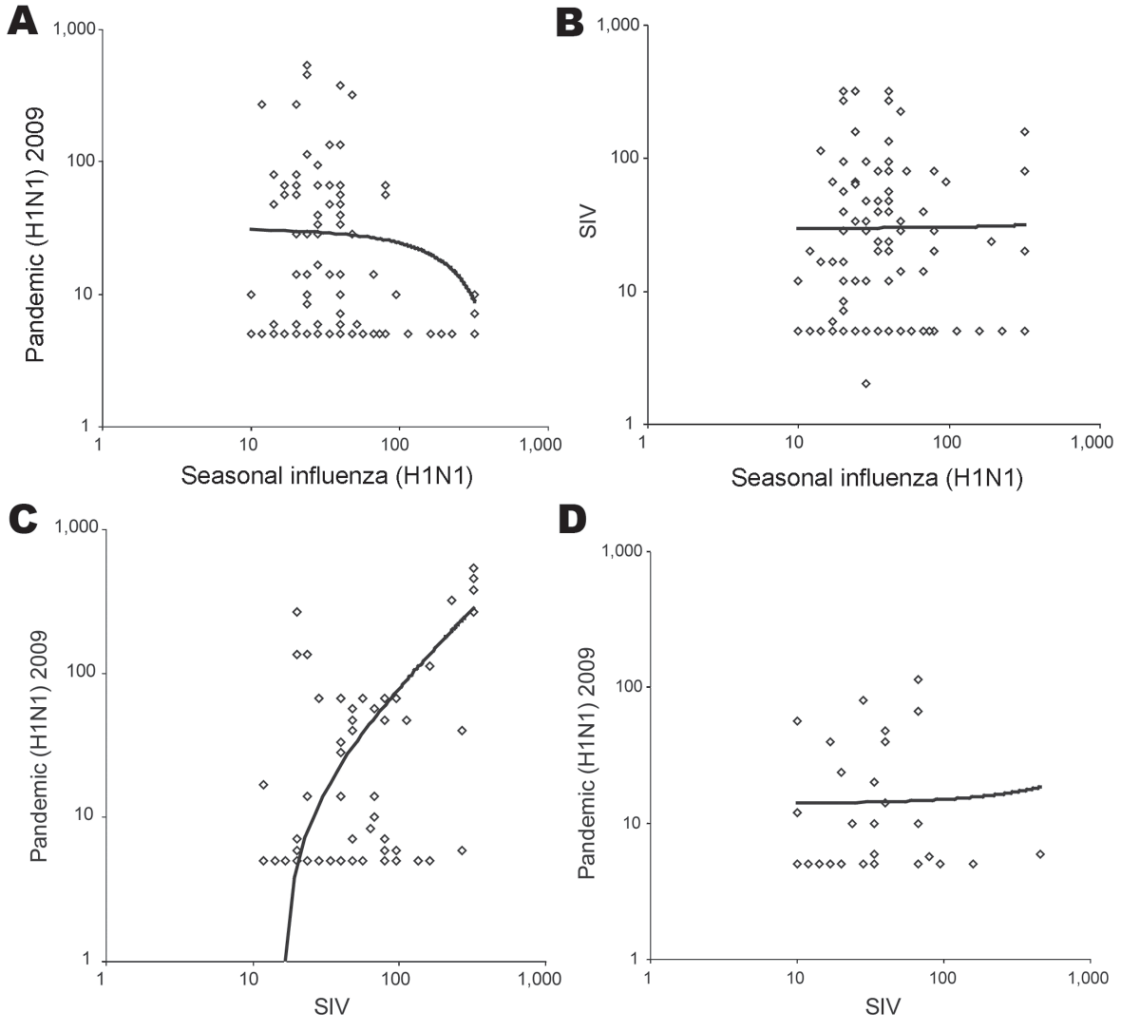
Geometric mean titers (>10) of antibodies against pandemic (H1N1) 2009 virus, seasonal influenza (H1N1) virus, and swine influenza virus of swine workers (A, B, C) and controls (D). Each symbol represents titer of 1 person; only persons with positive results (>10) for at least 1 of the 2 viruses of the panel are shown. Trend lines are shown; R^2^ values were R^2^>0.01 for panels A, B, and D and R^2^ = 0.5 for panel C.

Among SWs, being SIV positive increased the odds of being positive for pandemic (H1N1) 2009 virus by 2.4× (odds ratio [OR] 95% CI 1.3–4.3). Among controls, these chances were increased by 6× (OR 95% CI 2.9–12.6).

### Seasonal Influenza Virus Compared with Pandemic (H1N1) 2009 Virus and SIV

GMTs for seasonal influenza virus were significantly higher among SWs than controls (Table 2), and significantly more SWs than controls had antibodies against seasonal influenza virus, at least for titers ≥10 to 40 (Table 3). Among all age groups, more SWs than controls had antibodies against seasonal influenza (Table 4). GMTs among controls >60 years of age were significantly higher than those among younger controls (Table 2). Significantly more SWs and controls had antibodies against seasonal influenza virus than against pandemic (H1N1) 2009 virus and SIV (Table 3).

All SWs with antibodies against pandemic (H1N1) 2009 virus also had antibodies against seasonal influenza virus with the following exceptions: 1) GMTs were significantly higher for pandemic (H1N1) 2009 virus (50.8, 95% CI 37.7–68.4) than for seasonal influenza viruses (31.5, 95% CI 26.6–37.4) (p = 0.001); 2) GMTs of at least single positive serum samples did not correlate (R^2^<0.01, CC = 0.231; Figure, panel A); and 3) 17 of 21 samples with seasonal influenza virus titers >80 were negative for pandemic (H1N1) 2009 virus (cutoff <10). No correlation was found between GMTs of samples positive for seasonal influenza virus and SIV (R^2^<0.01, CC = 0.339; Figure, panel B). These results may indicate no substantial cross-reactivity between antibodies against pandemic (H1N1) 2009 virus or SIV and at least a recent seasonal influenza virus.

### Risk Factors

Odds of having antibodies against pandemic (H1N1) 2009 virus were 2.4× (95% CI 1.4–4.2) to 3.9 (95% CI 1.3–12) greater for SWs than for controls (cutoffs >10 to >160). Odds of having antibodies against SIV were 1.3× (95% CI 0.8–1.9) to 9.9 (95% CI 0.5–38.9) greater for SWs than for controls (cutoffs >10 to >80). Odds of being SIV positive were slightly higher for farmers (OR 2.3, 95% CI 1.1–5) than for slaughterhouse workers; odds of being positive for pandemic (H1N1) 2009 virus were only slightly higher for farmers than for slaughterhouse workers (OR 1.2, 95% CI 0.6–2.5). ORs for being positive for pandemic (H1N1) 2009 virus and for SIV were slightly higher for male SWs (1.7, 95% CI 0.8–3.5, and 1.1, 95% CI 0.6–2.3, respectively; cutoff >10). Among SWs, 26.5% self-reported receiving >1 dose of seasonal influenza vaccine during the past 5 years; among vaccinated SWs, the odds of having antibodies against pandemic (H1N1) 2009 virus (OR 1.3, 95% CI 0.6–2.6) as well as against SIV (OR 1.3, 95% CI 0.7–2.5; cutoff >10) were slightly higher than those for unvaccinated SWs. Odds of having antibodies against pandemic (H1N1) 2009 virus were slightly higher for SWs exposed to swine until the time of sampling (OR 1.5, 95% CI 0.7–3.3) in 2009 than for those who had no contact with swine after 2007. OR for having antibodies against SIV for SWs with pig contact until time of sampling was 0.5 (95% CI 0.2–1.1) compared with that for persons who had no contact after 1997. Thus, no significant associations were found between year of exposure and seroprevalence of antibodies against either virus.

## Discussion

At the time of blood collection from SWs (late July 2009), pandemic (H1N1) 2009 had spread to all continents, but intensity was still low in Europe, especially in Luxembourg and its neighboring countries. The only countries in which infection rates increased were the United Kingdom, Ireland, and Spain (where sporadic outbreaks occurred) (*25*). In 2009, Luxembourg had an intensive active surveillance system for influenza-like illnesses. Follow-up for all patients with suspected cases included patient travel history, RNA extraction, and PCR to detect pandemic (H1N1) 2009 virus. All patients with confirmed disease were monitored until at least early August. Patients and their contacts received prompt antiviral drug treatment, and home quarantine was recommended. In Luxembourg, ≈60 cases were reported and confirmed around the time that blood collection from SWs was ending. Until end of June 2009, almost all Luxembourg patients were epidemiologically unrelated, and the source of infection was not determined for one fifth (*26*). The first sustained transmissions were noted by mid September (J. Mossong, pers. comm.). The first cases of pandemic (H1N1) 2009 in swine on the European mainland were reported in January 2010 (*3*). Nevertheless, the difference in the time of blood collection from controls (December 2008) and from SWs (July 2009) is a limitation of our study.

The virus neutralization assay used measures neutralizing antibodies mainly against HA because antibodies were in the assay only during the virus entry phase (*20*). Nevertheless, we cannot exclude that residual antibodies against NA and M (93% and 98% aa identity between pandemic [H1N1] 2009 virus and SIV, respectively) may contribute to neutralization (*27*).

Because there is no correlate of protection for neutralizing antibodies or a definition of a positive titer measured by virus neutralization assay (*28*), we analyzed titers by using running cutoff values for positivity and compared GMTs. This analysis showed significantly higher prevalence of neutralizing antibodies against pandemic (H1N1) 2009 virus in SWs than in controls, and seropositivity decreased with age. Younger (<60 years) SWs had higher titers, and 2× more SWs than age-matched controls had neutralizing antibodies against pandemic (H1N1) 2009.

No evidence indicates that pandemic (H1N1) 2009 virus was present in swine in Europe in or before July 2009. Reactivity with pandemic (H1N1) 2009 virus correlated best with antibodies against SIV. Although this correlation was highly significant among SWs with relatively high titers for SIV, no such correlation was found among controls, in whom antibody levels against SIV were low. We speculate that the difference between the cohorts may reflect cross-reactive antibodies to another influenza virus more similar to SIV (with or without a minor contribution of antibodies against seasonal influenza) in SWs, in contrast to low, mainly cross-reacting seasonal influenza virus antibodies in controls. Serologic cross-reaction between SIV and pandemic (H1N1) 2009 virus in pigs was recently reported (*22*). Our results also showed that reactivity with pandemic (H1N1) 2009 (or SIV) in either cohort cannot be explained by cross-reactivity with a recent seasonal influenza virus used in this study. Nevertheless, because more SWs than controls were exposed to seasonal influenza virus, we cannot exclude the possibility that antibodies to pandemic (H1N1) 2009 virus or to SIV in the SWs may be caused by a more complex history of exposure to seasonal influenza virus of subtype H1 or to subclinical infections with pandemic (H1N1) 2009 virus during the first months of the pandemic.

Our finding of low levels of neutralizing antibodies against pandemic (H1N1) 2009 in controls (general population) is in agreement with findings of previous studies (*29*). Our findings that titers were less common but higher for older controls contrast with reports from the United Kingdom and Finland (*16**,**17*) but agree with findings of 2 studies in China, where elderly persons (≥60 years) had few or no neutralizing antibodies against this virus (*30**,**31*).

Our study also showed significantly higher prevalence of neutralizing antibodies against SIV in SWs than in the controls at cutoffs >20 to >160, but differences in GMTs were not significant. Similar serologic studies in humans in the United States showed markedly elevated antibody titers for North American SIVs of subtype H1N1 and H1N2 in SWs compared with controls (*5**,**8**,**10**,**11**,**32**,**33*). These studies used hemagglutination inhibition instead of virus neutralization assays and reported ORs for increased serologic responses instead of seroprevalence rates. The reported ORs, however, seem to be higher than those in our study (*8**,**32**,**33*) and could be partially explained by exclusion of persons with swine exposure in the US control groups.

Most persons undergo sequential infections with multiple antigenic variants of human influenza subtype H1N1 and H3N2 viruses throughout their lives. Such infections strongly increase the odds for serologic cross-reactions with antigenically distinct H1 viruses, as documented in experimental studies with pigs (*22*), and may explain why older persons in the general population have higher antibody titers to SIV than their younger counterparts. Both older and younger controls are unlikely to have been infected with SIV, but older persons have been exposed to a wider variety of human seasonal influenza viruses. This exposure is also reflected by a significant difference in GMTs for recent seasonal influenza virus in older than younger controls. In Luxembourg, elderly persons may have had contact with swine because during 1920–1947 in Luxembourg, 50%–22% of all households kept >5 pigs, but before 1979, there was no apparently substantial swine influenza activity in this part of Europe (*14*). Apart from antibodies to SIV, a few controls also had antibodies to pandemic (H1N1) 2009 virus, but these did not correlate with each other, suggesting a different cross-reactivity pattern than that for SWs. These findings show that in the absence of paired serum samples, presence of neutralizing antibodies to a given influenza virus does not necessarily reflect infection with that virus. Elevated antibody titers to SIV in part of the SWs may have resulted from exposure to the virus, but further studies are required to determine all possible causes.

In conclusion, titers of antibodies against pandemic (H1N1) 2009 virus and against an avian-like subtype H1N1 influenza virus were found more frequently and were higher for SWs than for controls. These titers cannot be explained by cross-reactivity with antibodies from recent seasonal influenza viruses. Neutralizing antibodies to both subtype H1N1 viruses showed some degree of correlation.

Further studies are needed to determine incidence of zoonotic SIV infections and the extent to which serologic responses correlate with infection. Neutralizing antibodies should confer at least partial protection against infection, reducing the risk that the avian-like subtype H1N1 SIV will cause major outbreaks of disease in humans.
